# Extracellular vesicles in female reproduction: from basic research to application

**DOI:** 10.1590/1984-3143-AR2025-0049

**Published:** 2025-08-05

**Authors:** Camila Azzolin de Souza, Gislaine dos Santos, Schaienni Fontoura Saldanha, Luca Angi Souza, Juliano Coelho da Silveira

**Affiliations:** 1 Departamento de Medicina Veterinária, Faculdade de Zootecnia e Engenharia de Alimentos – FZEA, Universidade de São Paulo – USP, Pirassununga, SP, Brasil; 2 Departamento de Reproducción Animal, Instituto Nacional de Investigación y Tecnología Agraria y Alimentaria – INIA, Agencia Estatal Consejo Superior de Investigaciones Científicas – CSIC, Madrid, España

**Keywords:** extracellular vesicles, female reproductive physiology, biomarkers, EVs supplementation, engineered EVs

## Abstract

*In vitro* embryo production (IVEP) offers an alternative approach for fertility preservation, genetic improvement, and reproductive research. However, *in vivo*, the female reproductive tract constitutes a dynamic microenvironment that undergoes critical changes crucial to support oocyte maturation, fertilization and embryo development. During IVEP, the absence of maternal-gamete and later maternal-embryo cross-talk can compromises both fertility and embryo development as well as quality. Extracellular vesicles (EVs) derived from the maternal reproductive tract, such as those from follicular fluid, oviductal fluid and uterine fluid, have attracted increasing attention due to their ability to carry bioactive biomolecules and partially restore this bidirectional communication when supplemented during IVEP. Moreover, EVs hold the potential to serve as indicator of the physiological or pathological state of reproductive structures as well as serving as real-time biomarkers. In addition, several studies suggest that EVs offer multiples advantages over conventional synthetics carries, opening new frontiers for modern drug or nucleotide delivery systems. Therefore, this review aims to provide a comprehensive overview of EVs derived from female reproductive tract, exploring their potential applications and challenges in enhancing IVEP outcomes and fertility treatments.

## Introduction

Assisted reproductive technologies constitute an essential tool in addressing human infertility, which affects approximately 186 million people globally (Inhorn and Patrizio, 2015). Additionally, in the context of animal reproduction, these biotechnologies offer significant potential to accelerate genetic improvement and enhance the productive efficiency of herds (Mello et al., 2016). However, the success of these techniques is directly linked to their ability to mimic the natural process that occurs in the organisms. Although several decades have passed since the birth of the first calf derived from in vitro production, embryos produced in vivo continue to demonstrate superior quality compared to those produced in vitro, resulting in a higher number of live births (Brackett et al., 1982; Sirard and Blondin, 1996).

Therefore, researchers are actively exploring strategies to improve the efficiency of in vitro embryo production to better replicate in vivo conditions aiming to establish a similar physiological environment. In this context, EVs have gained attention as a promising tool, due to their capacity to carry bioactive molecules, such as nucleic acids, metabolites and proteins, as well as due to their pivotal role in mediating intercellular communication (Valadi et al., 2007).

Moreover, EVs are synthesized and secreted by various regions of the female reproductive tract, and their cargo can be dynamically monitored, reflecting the physiological and pathophysiological states of their cells of origin. Owing to these properties, EVs have been recognized as reliable biomarkers for reproductive disorders (Bernardi and Balbi, 2020; Esfandyari et al., 2021). Another promising application of EVs is their use as drug delivery vehicles. EVs offer several advantages over synthetic nanoparticles, such as their unique protein-decorated phospholipid membranes which contain specific barcodes that can recognize targets cells both locally and at distance sites (specificity), they are inherently safer, exhibit superior bioavailability, possess natural targeting capability, and have the potential to efficiently transport biomolecules across biological barriers (Herrmann et al., 2021). Therefore, this literature review aims to summarize the know functions of EVs in the female reproductive tract, and explore their potential applications in the development of innovative strategies to enhance animal reproduction.

## Extracellular vesicles

EVs are nanoparticles that are released either constitutively or in response to specific stimuli by cells into the extracellular environment (Tannetta et al., 2014). They are considered mediators of cellular communication, as they carry bioactive molecules such as proteins, lipids, and nucleic acids (Simpson et al., 2008; Subra et al., 2007; ValadI et al., 2007). These EVs were described in biological fluids such as follicular fluid, cerebrospinal fluid, blood, urine, oviduct fluid, and uterine fluids (Burns et al., 2016; Cleys et al., 2014; Silveira et al., 2012; Lopera-Vasquez et al., 2016; Ng et al., 2013; Prunotto et al., 2013; Street et al., 2012). Furthermore, EVs can be found in the culture medium of different cell types, including media conditioned by bovine embryos produced in vitro (Bridi et al., 2021), as well by human embryos (Veraguas et al., 2021).

**Figure 1:**
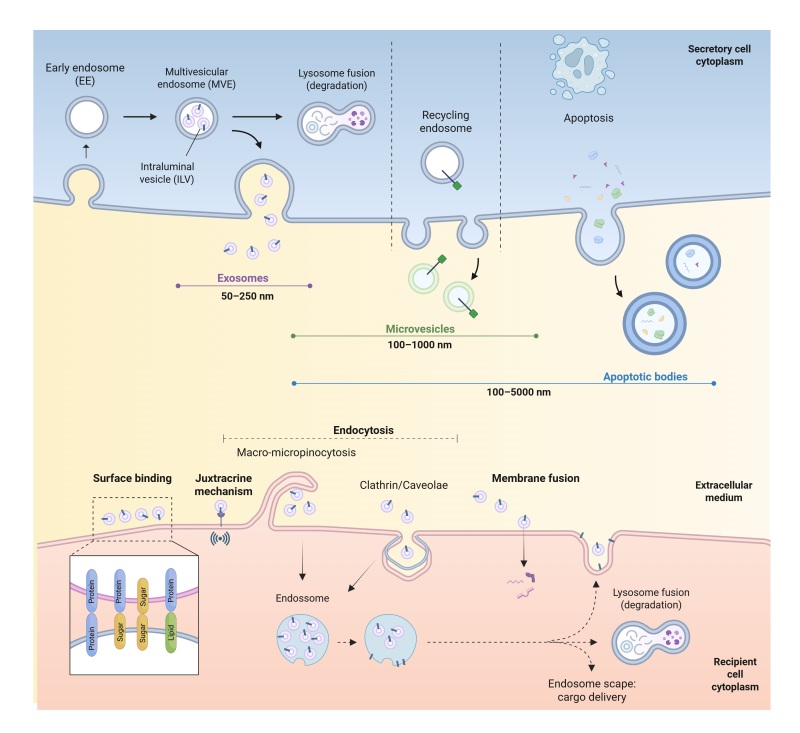
Biogenesis and secretion of EVs. The figure illustrates the biogenesis of exosomes, microvesicles, and apoptotic bodies within a secreting cell. It then shows the release of these particles into the extracellular environment and the size range of particles that can be found there. Finally, the figure depicts the uptake of EVs by recipient cells. This figure was created with BioRender (2025).

These nanoparticles can be classified into apoptotic bodies, microvesicles, or exosomes according to their biogenesis and size (György et al., 2011). Apoptotic bodies are generated from cell apoptosis and vary in size between 100 nm and 5000 nm. Microvesicles, on the other hand, are derived from the budding of the plasma membrane and range from 100 nm to 1000 nm in size. Finally, exosomes are 30 nm to 150 nm in size, originating from endosomal compartments (Mathivanan et al., 2010) (Figure 1). According to the MISEV 2023 guidelines, it is recommended to use the term “extracellular vesicles (EVs)” as a general term encompassing all types of vesicles released by cells, including exosomes, microvesicles, and apoptotic bodies (Welsh et al., 2024), followed by a good description of the methods used to separate the vesicles as well as characteristics such as protein markers, size and morphology.

During EVs’ biogenesis (Figure 1), intraluminal vesicles (ILVs) are formed and accumulate inside early endosomes. Multivesicular bodies (MVBs), or late endosomes, are formed following a maturation process. At this stage, the targeting of bioactive molecules to MVBs begins through the Endosomal Sorting Complex Required for Transport (ESCRT)-dependent pathway (Colombo et al., 2013; Stuffers et al., 2009). These family of proteins (ESCRT-0, -I, -II and -III) regulate cargo targeting into MVBs and the formation of ILVS, and the presence of other ESCRT proteins or ESCRT-accessory molecules, such and Alix, CD63 and TSG101, have often been offered as proof of their MVB origin (Mathieu et al., 2019). After the bioactive molecules are internalized into the EVs, the MVBs either join the lysosomes and undergo degradation or fuse with the plasma membrane to release their content into the extracellular environment. Although the mechanism involved in this process is not fully understood, studies have shown that the activation of RAB7 can induce the fusion of MVBs to lysosomes, and that RAB27 is involved in their fusion with the cell membrane (Ostrowski et al., 2010; Vanlandingham and Ceresa, 2009). Furthermore, the release of EVs involves several proteins in the transport and fusion of MVBs with the cell membrane, including the SNARE protein family (Jahn and Scheller, 2006). Therefore, the biogenesis of EVs includes three main steps: ILV formation, prevention of MVB degradation, and fusion of MVBs with the cell membrane. Once released into the extracellular space, EVs can reach recipient cells, interact with their cell membranes, and transfer their components (Van Niel et al., 2018).

The mechanism by which EVs determine which cell to enters remains incompletely characterized. EVs can target receptor cells through macropinocytosis or micropinocytosis, or through specific molecular interactions involving proteins, sugars, and lipids present in the cell membrane. One mode of signaling involves EVs using a juxtracrine mechanism, where they interact with receptors on the cell surface, inducing an intracellular response without delivering their content. Also, interaction can occur through the cleavage of EV membrane proteins, with the cleaved fragments binding to membrane receptors on the target cell to induce an intracellular response. EVs can also transmit enclosed molecules fusing with the cell membrane and releasing their contents inside the recipient cell (Prada and Meldolesi, 2016; Valadi et al., 2007). Finally, EVs can be internalized by endocytosis through different pathways including micropinocytosis, clathrin, and caveolae. Usually, the EVs are trafficked to early endosomes, where they may either be recycled back to the plasma membrane or degraded after fusion with the lysosome (Tian et al., 2014).

## The role of extracellular vesicles in the female reproductive tract

**Figure 2:**
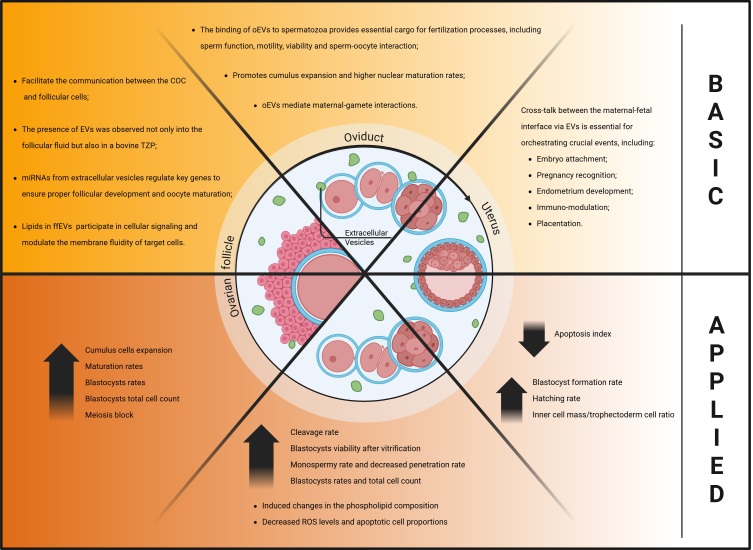
Extracellular vesicles and their role in female reproductive biology and their applicability in developing new strategies to improve animal reproduction. The figure illustrates at the top section the role of extracellular vesicles in the follicular, oviduct and uterine environment. The lower section of the figure demonstrates the results upon EVs supplementation at each stage of in vitro embryo production, replicating physiological process. This figure was created with BioRender (2025).

The establishment of pregnancy involves complex processes such as oogenesis, folliculogenesis, fertilization, early embryonic development, embryo hatching, adequate uterine conditions and conceptus implantation (Machtinger et al., 2016). During these processes, gametes and embryos are in constant communication with the components of the reproductive tissues and structures through endocrine, paracrine, and autocrine signaling (Kretser et al., 2002; Ávila et al., 2020). An important intercellular communication mechanism that has been increasingly recognized involves the secretion and uptake of EVs (Février and Raposo, 2004). The presence of EVs in the fluids of both the female and male reproductive tract, under physiological and pathological conditions, suggests their involvement in regulating key reproductive processes and their contribution to reproductive disorders. This also highlights their potential use as biomarkers. Additionally, their remarkable ability to transfer cargos between different cell types makes them excellent candidates as nanocarriers for target cell modulation (Qamar et al., 2020; Smith and Russell, 2022) (Figure 2).

### Extracellular vesicles derived from the ovarian follicle

EVs are present in both small (3-5 mm) and large (>9 mm) antral bovine follicles suggesting their participation in folliculogenesis and oogenesis (Hung et al., 2015). EVs were first described in the follicular fluid of mares and later identified in cows, woman, pigs, cats, buffalo, and rhino (Capra et al., 2022; Silveira et al., 2012; Ferraz et al., 2020; Gad et al., 2024; Matsuno et al., 2019; Santonocito et al., 2014; Sohel et al., 2013). The origin of EVs within follicular environment has been attributed to granulosa cells and cumulus-oocyte complexes (COCs) (Andrade et al., 2017). Consequently, EVs carry a variety of bioactive molecules crucial for regulating the follicular environment and facilitating communication between the COC and follicular cells (Ávila et al., 2020; Gabryś et al., 2024; Hung et al., 2015; Théry et al., 2002). Among the molecules carried by follicular fluid EVs (ffEVs) are proteins, such as enzymes and signaling proteins (Ferraz et al., 2020; Uzbekova et al., 2020). In cats, the proteins identified within ffEVs are predicted to be involved in pathways that regulate oxidative phosphorylation, extracellular matrix, actin cytoskeleton regulation, and tight junction integrity (Ferraz et al., 2020). In cows, the proteins of ffEVs from 3-5 mm follicles are predicted to be involved in endocytosis pathway and PI3K-Akt signaling pathways among others (Uzbekova et al., 2020). Therefore, the proteins present in ffEVs play a crucial role in regulating pathways associated with the follicular environment. Additionally, ffEVs carry nucleic acids such as mRNA, miRNAs, and other small non-coding RNAs (Navakanitworakul et al., 2016). The most studied nucleic acid in ffEVs are the miRNAs. MicroRNAs are small sequences of 20 to 24 nucleotides that play a crucial role in post-transcriptional regulation (Lee et al., 1993). Cells utilize these miRNAs to inhibit the translation of coding RNAs by inactivating or degrading the mRNA (Dykxhoorn et al., 2003). In cattle, specific miRNAs have been identified as critical regulators of pathways related to follicle development and oocyte maturation. For example, bta-miR-20a and bta-miR-494 are two validated modulators of PTEN, an important negative regulator of the PI3K-Akt signaling pathway. Higher levels of PTEN mRNA and lower levels of bta-miR-494 and bta-miR-20a were observed in follicular cells from follicles containing oocytes with poor quality, suggesting reduced activity of the PI3K-Akt pathway in these follicles (Andrade et al., 2017). These findings highlight the importance of miRNAs from EVs in ensuring appropriate follicular development and oocyte maturation by adjusting the expression of key regulatory genes. Lipids, including phospholipids and sphingolipids, are also present in ffEVs and participate in cellular signaling and modulate the membrane fluidity of target cells (Silveira et al., 2021). Using mass spectrometry, the lipid content of EVs from follicles containing oocytes with varying developmental stages (Non-CLEAVED, CLEAVED and BLASTOCYST) was analyzed. The results demonstrated that while most lipids were common across all groups, certain lipids, such as DAG 36:0 and PC 38:0, were unique to the BLAST group, suggesting a potential link to oocyte developmental competence (Silveira et al., 2021). The presence of EVs was observed not only into the follicular fluid but also within the bovine transzonal projections (TZP), using transmission electron microscopy (Marchais et al., 2022). This suggests that EVs are released from the TZP terminus and could possibly play a role in the direct communication between cumulus and oocyte.

### Extracellular vesicles derived from the oviduct

In mammals, the oviduct provides an essential environment for crucial events such as oocyte pick-up, final oocyte maturation, fertilization, preimplantation embryo development, and embryo transit to the uterus. These sequential events are supported by a dynamic oviductal fluid, which is formed by a selective transudate from the blood, secretory products from epithelial cells (oECs), and supplementation with follicular fluid upon ovulation (Aguilar and Reyley, 2005). Therefore, the composition of oviductal fluid includes ions, energy substrates, amino acids, proteins, prostaglandins, steroid hormones and growth factors (Leese et al., 2008). Recently, oviductal extracellular vesicles (oEVs) have emerged as another major component of interest, whose presence in the oviductal fluid was demonstrated in various species, including murine (Al-Dossary et al., 2013), porcine (Alcântara-Neto et al., 2020), bovine (Mazzarella et al., 2021), caprine (Mendes et al., 2024.), and humans (Bathala et al., 2018). Furthermore, oEVs have already been characterized *in vitro* through secretion by equine and canine oviductal cells spheroids (Lange-Consiglio et al., 2022, 2017).

Biogenesis of oEvs appears to be via the apocrine pathway, and evidence suggests that changes in the protein, mRNA, and ncRNA content occur across the stages of the estrous cycle, under action of hormonal control (Almiñana et al., 2018; Hamdi et al., 2021; Harris et al., 2020). Furthermore, the marked difference in content profile between oEVs and their parental epithelial cells indicates a selective packaging of cargo constituents (Anand et al., 2019; Fereshteh et al., 2018). Thus, this combined information suggests that oEVs are an important mechanism of cross-talk by transferring their cargo to gametes and embryos, modulating the oviductal environment according to physiological needs.

It has been demonstrated that oEVs can bind and fuse with the acrosomal membrane and midpiece of domestic cat spermatozoa, as well as with the sperm head, intermediate piece, and entire sperm body of porcine spermatozoa (Alcântara-Neto et al., 2020; Ferraz et al., 2019). In addition, it has been shown that important proteins related with male fertility, such as Ca^2+^- ATPase protein (PMCA4), can be delivered to spermatozoa via oEVs (Al-Dossary et al., 2013). In the absence of PMCA4, male mice are infertile due to inefficient sperm hyperactivation and motility (Okunade et al., 2004). Also, proteomics analysis from cats oEVs shows that these EVs contain important proteins involved in sperm motility, sperm-egg recognition, and sperm binding to zona pellucida (Ferraz et al., 2019).

The addition of oEVs with bovine frozen-thawed spermatozoa supports sperm survival, and stimulates processes associated with capacitation, increasing calcium levels as a second messenger for sperm function. (Franchi et al., 2020). Furthermore, the supplementation of oEVs during porcine *in vitro* fertilization significantly decreases polyspermy rates (Alcântara-Neto et al., 2020). Taken together, these findings suggest that the binding of oEVs to spermatozoa may enhance fertilization-related processes, including sperm function, motility, viability and sperm-oocyte interaction.

Physiologically, COCs are naturally exposed to follicular and oviductal fluid. Although multiple factor influence embryo development and quality, evidence in cattle suggests that the absence of key components present in these reproductive fluids during *in vitro* maturation of oocytes may contribute to suboptimal outcomes (Rizos et al., 2002). In this context, research aims to understand the role of oEVs during the final maturation of COCs. Canine oEVs derived from the culture of oviduct cells can be incorporated into COCs, and their supplementation during the *in vitro* maturation of COCs promotes cumulus expansion and higher nuclear maturation rates. Furthermore, supplementation activates the EGFR/MAPK signaling pathway in these COCs, a critical pathway for cumulus and oocyte maturation (Lee et al., 2020). The cargo content of oEVs secreted by multicellular canine spheroids shows enrichment for miR-30b, miR-375, and miR-503, which are essential regulators of follicular growth and oocyte maturation (Lange-Consiglio et al., 2017). Although these studies suggest an important role of oEVS in canine oocyte maturation, their complete contribution to the female gamete in other species remains unclear. Further research is needed to address gaps in female reproductive biology.

The early embryo development occurs inside the oviduct and, in cattle, during approximately 4-5 days the embryos stay in contact with the oviductal environment, that includes the oviductal fluid and consequently oEVs. The appropriate communication between the female reproductive tract and embryos is determinant for a successful pregnancy. In pregnant animals, the embryonic-maternal cross-talk, mediated by EVs, potentially initiated in the oviduct, and the presence of single embryo does not interfere with the concentration of oEVs, however it modulates the miRNA contents in oEVs and in oECs, in comparison with non-pregnant animals. These differentially expressed miRNAs modulate pathways, such PI3K/AKT, mTOR and MAPK, that are related to transcription, translation, proliferation, growth, control of the cytoskeletal organization, metabolism and survival (Mazzarella et al., 2021). In addition, the oEVs miRNA cargo are related to embryonic development, embryonic morphology and implantation. Further, oEVs contain mRNAs associated with epigenic DNA modification, indicating control of chromatin modification and epigenetic regulation during the embryo development (Almiñana et al., 2018; Bastos et al., 2022).

Additionally, the embryos successfully internalized *in vivo* oEVs, reinforcing their role as mediators of cell communication. oEVs were labeled with green fluorescent dye and were co-incubated with blastocysts with zona pelucida and hatching/hatched blastocysts. Confocal analysis revealed green fluorescence localized in the cytoplasm surrounding the nucleus of the blastocysts, both with and without the zona pelucida (Almiñana et al., 2017). Moreover, the supplementation with oEVs improved embryonic development and quality in terms of blastocysts rates, cell number, and hatching rates.

Furthermore, the supplementation of oEVs in IVC media over 8 days alters the embryonic transcriptome, potentially due to the incorporation of transcripts delivered by the oEVs into the embryo after uptake (Bauersachs et al., 2020). Once incorporated into the embryos, these transcripts can be translated into proteins that regulate embryonic gene expression. Another mechanism by which oEVs can regulate embryonic gene expression is through the delivery of miRNAs, since the oEVs miRNA have target genes in the embryos (Bauersachs et al., 2020).

In the same way, embryos can release EVs (eEVs), and their miRNA profile can be affected by the origin of these embryos (*in vivo* or *in vitro*) (Bridi et al., 2021). There is limited research on the effect of eEVs on oviductal cells; however, in mares, a previous study suggests that these EVs can be secreted by embryos and modulate the epithelium function through the transfer of early pregnancy factor (HSP10) and miRNAs (Bemis et al., 2012; Saadeldin et al., 2015; Tannetta et al., 2014). Furthermore, there is evidence that bovine oviduct epithelial cells adapt their transcriptome profile in response to the presence of embryos. Together, these finds suggest the existence of a real dialogue between the early embryo and oviduct, with EVs being strong candidates to mediate maternal-gamete or maternal-embryo interactions within the oviduct (Pavani et al., 2016; Schmaltz-Panneau et al., 2014).

### Extracellular vesicles derived from the uterus

The uterine cavity plays a crucial role in supporting the growth and development of the fetus. However, for this process to occur, an essential event must take place: the attachment of the conceptus and the development of the placental. In ruminants, this involves the interdigitation of trophoblast microvilli with the luminal epithelium in the caruncular and intercaruncular areas of the endometrium, extending throughout both uterine horns. In cattle, the majority of pregnancy losses occur during early embryogenesis or at the stages of conceptus implantation and the initial phases of placental development. Therefore, advancing our understanding of the physiological mechanisms involved in these events is vital for addressing pregnancy loss effectively (Davenport et al., 2023; Johnson et al., 2024; King et al., 1980).

It is well established that progesterone actions on the endometrium are required for conceptus elongation and the establishment and maintenance of pregnancy. This hormone stimulates the endometrial production of EVs (uEVs) and their release into the uterine lumen (Burns et al., 2018), which is crucial for communication between the cells within the implantation period and during the placenta development. In this sense, evidence suggests that the conceptus trophectoderm can take up uEVs from the uterine lumen, indicating a cross-talk between the maternal side and the conceptus. Additionally, uEVs can be taken up by endometrial epithelial cells, indicating a possible paracrine signaling mechanism (Burns et al., 2016). The endometrium can be modulated by uEVs secretion, responding differently to embryos produced through different methods, serving as a biosensor for their developmental potential (De Bem et al., 2024). Additionally, uEVs released by a disrupted endometrium have the potential to inhibit embryo development and suppress IFNT expression by altering MAPK signaling (Ichikawa et al., 2024).

As occurs in oviductal fluid, the uEVs also change their content profile across the estrous cycle under hormonal control, without changing their size and concentration. The higher number of upregulated miRNAs observed in uEVs in the stage 3 of estrous cycles (post-estrous period) coincides with embryo/conceptus development, reception, and implantation period, and the most abundant pathways regulate by these miRNAs involve important pathways to embryo development such as: MAPK signaling, Hedgehog signaling and Wnt signaling, indicating an important role of these nanoparticles in the establishment of pregnancy (Hamdi et al., 2021). In the same way, the protein profile within uEVs change between timepoints of the estrous cycles. Functional enrichment analysis of these proteins showed several pathways involved in regulation of endometrial physiology and early embryo development. Several of these pathways were activated at day 7 or day 16 of the estrous cycle compared to day 0, such as cell morphogenesis, cell adhesion, antioxidant activity, and cellular homeostasis. Furthermore, when the pooled of all stages of uEVs were supplemented to IVC medium it improves blastocyst rates, in comparison with non-supplemented zygotes (Piibor et al., 2023).

In cattle, non-invasive trophoblasts begin attaching to the uterine epithelium on day 19, while in sheep, this process occurs on day 16 (Nakamura et al., 2020). EV secreted by the conceptus (cEVs) are essential for conceptus implantation. Experiments conducted with ewe revealed that many differentially expressed proteins were found in EVs released by cEVs on days 17, 20 and 22 of pregnancy. Among these proteins, cEVs contain IFNT, and when bovine endometrial epithelial cells were supplemented with cEVs from days 20 and 22, the expression of adhesion molecules, such VCAM1, was up-regulated (Kusama et al., 2018). Additionally, in sheep, uEVs contain endogenous beta retroviruses (enJSRVs) and a several miRNAs that potentially affects the implantation process. When these uEVs carrying enJSRVs were supplemented to the conceptus, they stimulated trophectoderm cells to proliferate and secrete interferon-tau (INFT). INFT, is a crucial signal exclusively produced by the trophectoderm cells, necessary to maternal recognition of pregnancy (Beal et al., 2023; Burns et al., 2014; Ruiz-González et al., 2015).

In addition to the classical mechanisms of maternal pregnancy recognition, new insights have been proposed, regarding immune tolerance in pregnancy mediated by EVs. Pregnancy is characterized by a higher level of circulating EVs that non-pregnant state (Nair and Salomon, 2018). It has been demonstrated that PKH26-labeled EVs can cross the uterine environment and be detected in the blood circulation, after intrauterine infusion. These EVs, once recovered, can be taken up by neutrophils from naïve animals, *in vitro.* These findings suggest that placental-derived extracellular vesicles (pEVs) may contribute to the immune response during early pregnant development (Farias Fiorenza et al., 2024; Zhang et al., 2020). Furthermore, synchronized communication between the placenta and uterus is vital for successful survival of the conceptus, both sides exchange EVs as part of this complex dialog (Rosenfeld, 2024). Placenta extracellular vesicles (pEVs) carry large amounts of important cargo, such as vasoactive proteins. In humans, these proteins can influence the maternal vasculature and are involved in the pathogenesis of preeclampsia (Nair and Salomon, 2018). Moreover, angiogenesis is an indispensable biological process preparing the endometrium for implantation and placentation by providing a new vasculature. In the swine model, EVs released by trophectoderm cells and chorioallantois membranes contained abundant proteins and miRNAs, both were predicted regulators of angiogenesis-related pathways. The most abundant pro-angiogenic miRNAs found were miR-126-5p, miR-296-5p, miR-16 miR-17-5p (Bidarimath et al., 2017).

Overall, all these findings support that the cross-talk between the maternal-fetal interface via EVs is essential for orchestrating crucial events, including embryo attachment, pregnancy recognition, endometrium development, immuno-modulation and placentation.

## Potential applications of extracellular vesicles as biomarkers to predict female reproductive performance

Reproductive efficiency depends on the application of early and highly accurate diagnostic methods, which allow for the identification of factors that compromise fertility. This integrated approach enables the adoption of effective interventions, both in field management practices and in the use of assisted reproduction techniques, contributing to the maximization of conception rates and the minimization of reproductive losses.

The regulation and occurrence of different physiological events are critically influenced by complex cellular interactions (Di Pietro, 2016). In the reproductive context, the success of pregnancy depends on processes such as gametogenesis, embryonic development, maternal recognition of pregnancy, among others. For these processes to occur efficiently, the cells involved employ various communication mechanisms. This includes, among other strategies, direct contact through GAP junctions and the secretion of extracellular signals, encompassing both the short-range paracrine signaling mediated by molecules such as cytokines and chemokines, as well as long-range endocrine signaling performed by secreted hormones (Di Pietro, 2016; Machtinger et al., 2016). Building upon this concept, EVs have been suggested as a novel mechanism for mediating cell communication between tissues. These vesicles reflect the environmental conditions through their molecular contents, making them a potential tool for assessing reproductive health (Al-Dossary et al., 2013; Silveira et al., 2018; Ruiz-González et al., 2015). EVs have the ability to exert both local and systemic effects and are involved in a wide range of biological functions, in both physiological and pathological conditions (Bastos et al., 2022; Lin and Gregory, 2015). Based on their functional versatility, EVs have been widely explored in various studies as a diagnostic tool. Their potential applications range from cancer diagnosis (Castillo et al., 2018) and metastatic potential assessment (Li et al., 2020) serving as indicators of the health of the urogenital system (Barreiro et al., 2021). In the reproductive field, small EVs have been identified in various biological fluids, including follicular fluid (Silveira et al., 2012), oviductal fluid (Mazzarella et al., 2021), secreted in culture media (Bridi et al., 2021) and in uterine flushing (Leal et al., 2022).

Given their role in cell-to-cell communication and their ability to reflect physiological conditions, EVs have emerged as promising biomarkers in reproductive biology. This review will highlight their potential as diagnostic tools in key reproductive events, discussing their applications in assessing fertility, embryonic development, and overall reproductive health.

### Follicle environment

The ovarian follicle is a dynamic microenvironment within the ovary, composed of theca cells, granulosa cells, cumulus cells, and the oocyte (Knight and Glister, 2006). Follicular growth is a highly regulated process that involves the proliferation of somatic cells and the accumulation of follicular fluid, which originates from plasma exudate and contains a variety of biomolecules, including hormones and metabolites (Edson et al., 2009). This environment plays a crucial role in supporting oocyte maturation and orchestrating the cellular and molecular events required for successful folliculogenesis. In this context, EVs have emerged as key mediators of intercellular communication, facilitating the exchange of molecular signals between follicular cells (Andrade et al., 2017). Several studies have successfully isolated EVs from follicular fluid in various species, including equine, bovine, and human. Notably, small EVs were first described in the follicular fluid of mares, highlighting their potential role in follicular physiology and oocyte competence (Silveira et al., 2012).

EVs in follicular fluid have been identified as potential biomarkers of ovarian pathophysiology. Notably, studies have reported differential expression of miRNAs in follicular EVs from healthy women compared to those diagnosed with polycystic ovary syndrome (PCOS), suggesting that alterations in EV molecular cargo may reflect underlying pathological dysregulation (Rooda et al., 2020). Additionally, variations in the miRNA profiles of follicular EVs have been associated with fertilization success and embryo quality (Machtinger et al., 2017; Martinez et al., 2018). These findings highlight the potential of EVs as diagnostic tools for reproductive competence. However, despite advancements in EV characterization and promising evidence supporting their relevance to oocyte competence, their application as a routine diagnostic method remains challenging. A major limitation is the invasive nature of follicular fluid collection, which restricts its feasibility for widespread clinical use.

Therefore, in the context of oocyte maturation, follicular fluid EVs have been investigated as bioactive agents with potential applications in assisted reproductive technologies. Notably, EVs from bovine follicular fluid of both small (3–5 mm) and large (>9 mm) follicles have been shown to induce cumulus expansion during in vitro maturation (Hung et al., 2015). Their therapeutic potential in reproductive biotechnologies will be further explored in a later section.

### Oviduct environment

The oviduct plays a crucial role in reproduction, providing the environment for fertilization and early embryonic development. Its secretions contain a wide range of bioactive molecules, including growth factors, metabolic regulators, and immune-related proteins, which contribute to oocyte competence and embryo quality (Aviles et al., 2010). Among these components, EVs have been identified as key mediators of intercellular communication in the oviductal microenvironment. Initially described in murine models and termed “oviductosomes” (Al-Dossary et al., 2013), these vesicles carry molecular cargo that reflects the physiological conditions of the reproductive tract. Several studies have characterized specific miRNAs in oviductal EVs as potential indicators of embryo viability and developmental competence (Lopera-Vasquez et al., 2016). Despite the promising role of oviductal EVs in reproductive processes, their application as diagnostic tools remain impractical, particularly in humans, due to the anatomical constraints of the oviduct, which is a narrow and delicate structure that makes fluid collection unfeasible. Nevertheless, the characterization of their molecular cargo provides valuable insights into the factors influencing embryo quality and development. Future research may focus on identifying key bioactive molecules within these vesicles that could be incorporated into engineered EV-based supplements, offering potential applications in assisted reproductive technologies.

### Endometrial-conceptus cross-talk

The communication between the embryo and the endometrium is crucial for pregnancy success, as both must be precisely synchronized to create an environment conducive to implantation. The embryo development depends on endometrial secretions, collectively known as histotroph, which provide essential nutrients, growth factors, and signaling molecules necessary for early development (Forde and Lonergan, 2012). Simultaneously, the maternal endometrium must reach an optimal receptive state, a transient phase known as the window of implantation (WOI), during which epithelial cells undergo structural and molecular modifications to support embryo attachment and invasion (Hart et al., 2022). A key aspect of this interaction is the embryo’s ability to signal its presence to the maternal system, preventing luteolysis and ensuring continued progesterone production. In ruminants, this function is largely mediated by interferon tau (IFNT), a cytokine secreted by the conceptus that regulates the maternal immune response and maintains corpus luteum function (Hansen et al., 2017). Recent evidence suggests that EVs play a critical role in this signaling process (Burns et al., 2016; Ng et al., 2013). EVs isolated from uterine flushings of cows and sheep during the peri-implantation period have been shown to activate interferon-stimulated genes in primary endometrial epithelial cells (Kusama et al., 2018; Nakamura et al., 2016). Since these EVs originate from both embryonic and maternal sources (Nakamura et al., 2016), they may act as carriers of molecular signals that mediate IFNT effects on the endometrium.

Beyond their role in maternal recognition of pregnancy, EVs actively contribute to key implantation processes. Experimental models have demonstrated that EVs derived from estradiol- and progesterone-primed human endometrial adenocarcinoma ECC-1 cells (Greening et al., 2016), as well as EVs secreted by human primary decidual stromal cells (Liu et al., 2020), enhance embryo hatching, outgrowth, and implantation success in mice (Gurung et al., 2020). These findings reinforce the concept that EVs facilitate embryo-endometrium communication, modulating implantation efficiency.

The production and composition of EVs are also influenced by hormonal changes, further supporting their role in the uterine environment (Greening et al., 2016). In sheep, EV concentration increases between Days 10 and 14 post-estrus, with progesterone administration further stimulating their release, whereas the use of a progesterone receptor antagonist suppresses EV secretion (Burns et al., 2018). Similarly, in bovines, EV size and abundance vary according to embryo quality, suggesting a potential role in assessing embryo viability (Dissanayake et al., 2020; Melo-Baez et al., 2020). Additionally, miRNA profiling of EVs collected at different pregnancy stages has revealed dynamic shifts in miRNA expression, with miR-98 emerging as a key regulator of maternal immune adaptation. Notably, synthetic miR-98 has been shown to alter gene expression in bovine endometrial epithelial cells, reinforcing the role of EVs in modulating the maternal environment to favor pregnancy establishment (Nakamura et al., 2019).

Given the substantial evidence supporting the involvement of EVs in embryo-endometrial communication and implantation, these vesicles have emerged as potential biomarkers of both embryo quality and uterine receptivity. Due to their capacity to transport bioactive molecules, EVs may also serve as indicators of the endometrial environment’s receptivity status (Hart et al., 2022; Homer et al., 2017). However, uterine receptivity can be compromised by various conditions, including chronic endometritis (Kuroda et al., 2020), obesity (Bellver et al., 2013), endometriosis (Lessey and Kim, 2017), and uterine malignancies, all of which disrupt the endometrial microenvironment and impair embryo-maternal interactions. These findings have generated growing interest in EVs as potential non-invasive biomarkers of uterine receptivity. Currently, endometrial receptivity assessment in human patients is subjective and invasive. Gene expression analyses of endometrial biopsies, performed using commercially available kits such as ERA-Test and beREADY, were initially expected to personalize embryo transfer decisions (Garratt and Rahmati, 2023). However, these methods are limited by the dynamic heterogeneity of endometrial cells, as dominant cell type ratios fluctuate throughout the menstrual cycle and may be influenced by disease states (Lessey and Kim, 2017). Consequently, gene expression variations in bulk endometrial samples likely reflect both shifts in cellular composition and intrinsic transcriptional changes within individual cell types.

EVs, by contrast, offer a compelling alternative as a minimally invasive tool for assessing both physiological regulation and pathological dysregulation in the endometrial environment. As they co-express markers from their cell of origin alongside functional biomolecules, EVs could serve as real-time indicators of uterine receptivity (Galio et al., 2023; Li et al., 2021). Notably, hsa-miR-30d has been identified as the most differentially expressed EV-associated miRNA in endometrial fluid during the window of implantation, reinforcing its potential as a biomarker of receptivity (Altmäe et al., 2013; Vilella et al., 2015). Given their ability to dynamically reflect the uterine microenvironment, EVs emerge as promising candidates for a “liquid biopsy” approach. Their molecular characterization could provide a minimally invasive strategy for predicting implantation success and diagnosing endometrial dysfunctions, representing a significant advancement in fertility assessment and reproductive medicine

### *In vitro* production

Beyond their role in embryo-maternal communication, EVs also hold great potential for improving *in vitro* embryo production. EVs are naturally secreted into the embryo culture medium, offering a valuable and currently underutilized resource for non-invasive assessment of embryo quality (Dissanayake et al., 2020; Pavani et al., 2020). Traditional embryo selection relies primarily on morphological evaluation, sometimes complemented by invasive embryo or trophoblast biopsies for genetic screening (Anagnostopoulou et al., 2022). However, morphology alone has limited predictive power, as embryos with similar appearances can have significantly different developmental potentials.

The embryo secretome, including EVs, has emerged as a promising substrate for identification of reliable biomarkers of developmental competence. Studies have shown that embryos with higher implantation potential tend to release fewer EVs into the culture medium on Days 3 and 5 of development compared to embryos that fail to implant, suggesting that EV quantity itself may serve as an indicator of embryo viability (Abu-Halima et al., 2017), corroborating similar findings reported in bovines (Dissanayake et al., 2020; Mellisho et al., 2017). Additionally, the molecular cargo of EVs, including proteins, mRNAs (Giacomini et al., 2021), and miRNAs (Bridi et al., 2021), lipids and metabolites (Gatien et al., 2019), could provide deeper insights into embryo fitness and implantation potential. First evidence of the functional role of embryo-derived EVs in assisted reproduction techniques was provided by the co-culture of porcine parthenogenetic embryos and embryos generated by somatic cell nuclear transfer (SCNT), where EVs carrying pluripotency-related transcripts (*OCT4*, *SOX2*, *C-MYC*, and *NANOG*) improved the developmental competence of SCNT embryos (Saadeldin et al., 2014). Melo-Baez et al (2020) provided evidence that the miRNA content of EVs reflects embryo developmental competence. In this study, the EVs contained upregulated bta-miR-103, bta-miR-502a, bta-miR-100, and bta-miR-1, as well as downregulated bta-miR-92a, bta-miR-140, bta-miR-2285a, and bta-miR-222 were secreted by embryos that reached the blastocyst stage compared to those arrested at the 8–16-cell stage (Melo-Baez et al., 2020). Based on these findings, the authors suggested that the wide variety of miRNAs can be found within the EVs could serve as indicators of embryo quality, and that the detection or manipulation of specific miRNAs may contribute to improving in vitro embryo production systems.

Another major challenge in assisted reproductive technologies (ART) is determining the optimal timing for embryo transfer. Since EVs dynamically reflect the physiological state of embryos, their characterization may help refine the timing of intrauterine transfer, potentially improving pregnancy rates. Given that liquid biopsy technologies based on EVs are already being explored in oncology (Castillo et al., 2018), similar approaches could be adapted for ART, transforming embryo selection and transfer strategies into more precise, non-invasive procedures. By harnessing EV-based diagnostics, in vitro embryo production could become more efficient, ultimately enhancing the success of ART and improving clinical outcomes. Conventional *in vitro* fertilization (IVF) methods use Petri dishes or micro-wells, ensuring stability with oil and individual incubators. However, these systems are static and do not accurately replicate physiological conditions, as the embryo, in its natural environment, is exposed to mechanical and biochemical stimuli while traveling through the oviduct to the uterus (Fauci and Dillon, 2006). Furthermore, the frequent handling of embryos still contributes to the high cost and low efficiency of IVF cycles. In this context, microfluidics emerges as a promising technology, allowing precise control of small fluid volumes and creating a dynamic environment that closely mimics the physiological environment. Additionally, microfluidics enables real-time collection of culture media, which would allow the analysis of extracellular vesicles secreted by the embryo, creating a liquid biopsy in real-time (Greco et al., 2017). This approach could provide valuable insights into embryo quality, aiding in embryo selection and optimizing assisted reproductive techniques.

## Therapeutic applications of extracellular vesicles in *in vitro* embryo production systems

Outside the field of reproduction, the application of EVs in therapeutic formulations has already become a reality. For instance, during the COVID-19 pandemic, EXO-CD24 was successfully used to mitigate the inflammatory response triggered by COVID-19 infection (Shapira et al., 2022). In that sense we synthesized the experiments in the past years that tested the effect of EVs from maternal biological fluids that physiologically interact with the oocyte and/or embryo.

### Follicular fluid EVs

Since the discovery of extracellular vesicles (EVs) in equine follicular fluid by Silveira et al. (2012), their in vitro application has been explored through various strategies to improve IVP, differing in both origin and methods of application. In 2017, Silveira and colleagues investigated the effects of EVs derived from follicular fluid of small follicles (3–6 mm) and preovulatory follicles during in vitro maturation (IVM) of cattle oocytes and in vitro culture (IVC) of cattle embryos. Supplementation with EVs from 3–6 mm follicles altered global DNA methylation and hydroxymethylation levels while also enhancing blastocyst formation rates. The most observed outcome of ffEV co-incubation during IVM has been cumulus cell expansion (Hung et al., 2015; Gabryś et al., 2024) and increased maturation rates (Makieva et al., 2025; Gabryś et al., 2022). Other reported effects include meiotic arrest of oocytes (Pioltine et al., 2020) and an increase in the total cell count of blastocysts (Pérez-Garcia et al., 2025) (Table 1).

**Table 1:** COCs coincubation with ffEVs experiments in chronological and species order. Gene expression outcomes were not mentioned in this review.

**Target cell**1	**EVs origin**	**IVEP step of supplementation**2	**Functional outcomes**3	**Species**	**References**
COCs	Small and large follicles	IVM	Increased cumulus expansion	Bovine	Hung et al. (2015)
COCs	Follicular fluid from 3-6 mm follicles	IVM and IVC	Increased blastocysts rates	Bovine	Silveira et al. (2017)
COCs	Estrous cycle Stage 3 and 1 follicular fluid	IVM	EVs from distinct stages of the estrous cycle differently modulate genes within cumulus cells	Bovine	Ávila et al. (2020)
COCs	Follicular fluid from 3-6 mm follicles	IVM	Meiosis resumption inhibition	Bovine	Pioltine et al. (2020)
COCs	Follicular fluid from 3-6 mm follicles	IVM	Increased blastocysts total cell count	Bovine	Pérez-Garcia et al. (2025)
COCs	Follicular fluid from 3-6 mm follicles	IVM	Increased maturation rates	Human	Makieva et al. (2025)
Compacted COCs	Follicular fluid from 3-6 mm follicles	IVM and IVC	Increased maturation rates	Equine	Gabryś et al. (2022)
Compacted and expanded COCs	Follicular fluid from 3-6 mm follicles	IVM	Increased cumulus expansion. Viability increased in compacted COCs, but decreased in expanded compared to the controls	Equine	Gabryś et al. (2024)

^1^Target cell: COCs: cumulus-oocyte complex. ^2^IVEP step supplementation: IVM: *in vitro* maturation; IVC: *in vitro* culture. ^3^Functional Outcomes: EVs: extracellular vesicles.

### Oviductal EVs

After being released from the ovary, the oocyte passes through the oviductal infundibulum and remains in the ampulla until the arrival of spermatozoa for fertilization. Numerous studies have investigated the effects of oviductal EVs on the conditioning of oocytes, spermatozoa, and mainly embryos. In bovines, the co-incubation of oEVs with embryos was shown to lead to increased blastocysts viability after vitrification (Lopera-Vasquez et al., 2017) and changes in the embryo phospholipid composition (Banliat et al., 2020). In porcine, the oEVs were found as an alternative to decrease the polyspermy but not drastically the penetration rate (Alcântara-Neto et al., 2020), also it induced an increase in cleavage rate (Alcântara-Neto et al., 2022), blastocysts rates and total cell count (Fu et al., 2022) (Table 2).

**Table 2:** COCs and embryos coincubation with oviductal fluid EVs experiments in chronological order. Gene expression outcomes were not mentioned in this review.

**Target cell**	**Supplemented EVs origin**	**IVEP step of supplementation**1	**Functional outcomes**2	**Species**	**References**
Zygotes	Oviductal fluid Isthmic portion	IVC	Isthmic EVs increased blastocysts viability after vitrification depending of EVs isolation protocol	Bovine	Lopera-Vasquez et al. (2017)
Zygotes	Oviductal fluid	IVC	Induced changes in the phospholipid composition of resulting embryos	Bovine	Banliat et al. (2020)
Oocytes	Oviductal fluid	IVF	Increased monospermy rate	Porcine	Alcântara-Neto et al. (2020)
			Decreased penetration rate		
Zygotes	Oviductal fluid	IVC	Two days of EVs treatment increased cleavage rate	Porcine	Alcântara-Neto et al. (2022)
Two-cells murine embryos	Oviductal fluid of Endometriose and health patients	IVC	Endometriose's EVs increased ROS levels, decreased MMP and increased apoptotic index compared to non-endometriose EVs	Human EVs	Li et al. (2023a)
				Murine embryos	
			Non-endometriosis EVs increased blastocysts rates compared to no EVs supplementation and Endometriose EVs supplementation.		
Two-cells murine embryos	Oviductal fluid	IVC	Increased blastocyst rate, hatching rate, as well as total cell number of blastocysts	Human EVs	Li et al. (2023b)
				Murine embryos	
			Decreased ROS levels and apoptotic cell proportions		
*In vivo* embryos	Oviductal fluid	IVC	Significant downregulation of ROS and 5-methylcytosine (5-mC)	Rabbit	Qu et al. (2020)
			Increased the blastocyst rate of embryos		
*In vivo* embryos	Oviductal fluid	IVC	Incubation of embryos with EVs had a positive effect at 48 h and 72 h, which disappeared by 96 h of IVC	Rabbit	Qu et al. (2022)
			EVs at a concentration of 9.1×10^12 particles/mL showed a negative effect at 96 h.		
			Replacement of EVs and medium during IVC could sustain embryonic development		
			Loss or gain of renewal in the IVC system affected EVs’ influence on embryo transcriptome, embryonic ROS, autophagy, epigenetic state and apoptosis		

^1^IVEP step supplementation: IVM: *in vitro* maturation; IVF: *in vitro* fertilization; IVC: *in vitro* culture. ^2^Functional outcomes: EVs: extracellular vesicles; ROS: reactive oxygen species. MMP: mitochondrial membrane potentials. IVC: *in vitro* culture.

The coincubation of oEVs from humans with endometriosis was found to be deleterious to murine embryos, by increasing ROS levels and apoptotic index, and decreasing MMP, when compared to the healthy oEVs coincubation (Li et al., 2023a). The oEV from health patients, on the other hand, decreased ROS levels and apoptotic cells proportions when compared to a control (Li et al., 2023b). These results highlight a possible resource to endometriosis treatment, also corroborating previous studies suggesting that EVs from endometriosis contribute to the pathophysiological process of angiogenesis and invasion (Khalaj et al., 2019). These findings also indicate the potential presence of a biomarker in EVs for early diagnosis. This could be crucial for improving disease prognosis (Shomali et al., 2020).

### Uterine EVs

A crucial stage of reproduction is embryo development during its implantation in the uterus. Throughout gestation, the fetus remains in the uterus, interacting with extracellular vesicles (EVs), suggesting that these vesicles may play a key role in pregnancy success. However, studies investigating the effects of uEVs through co-incubation with embryos remain limited.

Some experiments have reported an increase in blastocyst formation rates (Qiao et al., 2018; Piibor et al., 2023), along with a potential enhancement in hatching rates (Qiao et al., 2018; Aguilera et al., 2024). Additional findings suggest improvements in embryo quality, such as a higher inner cell mass/trophectoderm cell ratio and a reduced apoptosis index (Table 3).

**Table 3:** Embryos coincubation with uterine fluid EVs experiments in chronological order. Gene expression outcomes were not mentioned in this review.

**Target cell**	**Supplemented EVs origin**	**IVEP step of supplementation**1	**Functional outcomes**2	**Species**	**References**
4-day embryo	Uterine fluid	IVC	Increased the blastocyst formation rate, hatching rate, inner cell mass/trophectoderm cell ratio	Bovine	Qiao et al., 2018
2-day embryo	Uterine fluid	IVC	Luteal phase UF-EVs supplementation increased *in vitro* blastocyst rates	Bovine	Piibor et al., 2023
5-day embryos	Uterine fluid	IVC	Uterine EVs induced a sustained increase in diameter during embryonic development and a tendency towards a greater number of expanded and hatched blastocysts.	Bovine	Aguilera et al., 2024

^1^IVEP step supplementation: IVC: *in vitro* culture. ^2^Functional outcomes: UF-EVs: extracellular vesicles derivate from uterine fluid.

### Perspectives

Other approaches continue to be explored, including the combination of EVs from different sources (Table 4), the use of distinct isolation methods (Asaadi et al., 2021; Pérez-García et al., 2025), variations in EV dosage, the optimal IVEP supplementation stage, and EVs derived from different phases of the estrous cycle. The lack of standardized results, even when similar parameters are assessed, may be attributed to these variables, which remain challenging to standardize at this stage of our knowledge.

**Table 4:** COCs and embryos coincubation with mixed EVs experiments in chronological order. Gene expression outcomes were not mentioned in this review.

**Target cell**	**Supplemented EVs origin**1	**IVEP step of supplementation**2	**Functional outcomes**3	**Species**	**References**
Oocyte	Follicular and oviductal fluid (Isolated through UC and SEC)	IVM	Blastocyst yield and quality were higher in groups supplemented with EVs isolated from FF and OF by UC, with higher total cell numbers and a lower apoptotic cell ratio compared with other groups	Bovine	Asaadi et al. (2021)
Zygotes	Oviductal and uterus fluid	IVC	Higher survival rates after vitrification/warming Increased blastocysts total cell number	Bovine	Leal et al. (2022)
			Increased blastocysts total cell number		
			Decreased lipid content		

^1^Supplemented EVs origin: UC ultracentrifugation; SEC: size exclusion chromatography. ^2^IVEP step of supplementation: IVM: *in vitro* maturation; IVC: *in vitro* culture. ^3^Functional Outcomes: FF: follicular fluid; OF: oviductal fluid; UC: ultracentrifugation.

An innovative approach to overcome the above challenges has been explored through the use of synthetic vesicles, such as liposomes, and modified EVs. Engineered EVs represent a promising strategy that allows for the selection of their contents, particularly miRNAs.

## Engineered extracellular vesicles: how far are we?

Since it was discovered that EVs facilitate intercellular communication and that their cargo provides insights into the physiological or pathological status of several biological processes, there has been growing interest in leveraging them within fields like nanotechnology and nanomedicine as a natural tool for delivering bioactive molecules. Synthetic nanoparticles can be categorized on their chemical properties into organic, inorganic, and carbon-based types. Among the approved nanomaterials, polymeric liposomes are the most common (Namiot et al., 2023). Comparing EVs with artificial liposomes, both share a similar lipid bilayer structure, EVs demonstrate superior delivery efficiency due to their enhanced ability to enter recipient cells (van der Koog et al., 2022). Additionally, EVs exhibit low immunogenicity, innate stability, specificity for target cells and a remarkable natural capability to traverse biological barriers (Chen et al., 2021; Sahu et al., 2021). Most of the advantages of EVs in terms of cell specificity are due to the corona of proteins that are part of the lipid bilayer naturally inherited from the donor cells, thus strategies to mimic this natural ability are a challenge for synthetic vesicles.

Although, EVs are composed of a lipid bilayer that acts as a natural barrier, protecting their contents from degradation in the various biological fluids in which they are present. This characteristic also poses a challenge for the loading of exogenous molecules. To address this, several methodologies have been developed, which can be categorized into two major groups: cell based EVs-loading methods and non-cell-based methods (Mougenot et al., 2022). The main distinction between these groups lies in the fact that, in cell based EVs-loading methods, cells are directly transfected, and the EVs they secrete are loaded with the molecule of interest. In contrast, in non-cell-based methods, EVs are isolated from different cellular origins and subsequently loaded with the molecule of interest (Amiri et al., 2022; Vader et al., 2016). Different siRNA, miRNAs, proteins, CRISPR/ Cas9, hydrophobic compound derived natural product, and anticancer drugs can be loaded into EVs through sonication, electroporation, transfection reagents, and a specific buffer agent (Han et al., 2021).

In this sense, EVs can be loaded with miRNA for safe and efficient delivery to target cells. For instance, miR-124-3p has been successfully delivered to neuronal stem cells through umbilical cord blood mononuclear cell-derived EVs, playing a significant role in Parkinson’s disease due to its action on pro-neurogenic and neuroprotective pathways (Esteves et al., 2022). Another study achieved the delivery of interfering RNA to colorectal cancer cells through RAW 264.7 cell-derived EVs, modifying the tumor’s immunosuppressive microenvironment and promoting immune cell infiltration (Pei et al., 2021). One of the few studies conducted in the field of reproduction that utilizes this incorporation methodology, employed EVs derived from Sertoli cells to cross the blood-testis barrier and deliver a miR-24-3p inhibitor to germ cells, enhancing sperm motility. This could represent a potential tool for the clinical treatment of astenospermia (Chen et al., 2023). Despite EVs offering many advantages as a natural delivery tool, there are several challenges associated wither their use, such as the source of EVs, scalability and standardization of EV production, EVs purity, standardization of isolation methods, specific target delivery, loading capacity, dose selection, assessment, administration (route, frequency, time window), efficient and effective EV loading methods, as some methods may impact the stability of EVs (Claridge et al., 2021; Han et al., 2021).

To overcome some of the challenges associated with using biological fluid derived EVs as nanocarriers, recent studies have proposed the development of hybrid systems combining EVs and liposomes. Hybrids are expected to benefit from the natural properties of EVs, such as resistance to opsonization, better biological barrier crossing, better endosomal escape, and biocompatibility (Mougenot et al., 2022). Likewise, the hybrids inherit from the liposomes high percentual of drug encapsuled in the vector and loading capacity, and relatively easy engineering (Ducrot et al., 2023). The development of new delivery strategies using hybrid EVs may represent a promising tool for delivery systems, particularly in their applicability to animal reproduction—an entirely new area yet to be explored.

Taken together, the available evidence underscores the fundamental role of EVs in key processes related to reproductive biology. Despite significant advances, important knowledge gaps persist and require further investigation. The potential application of EVs as biomarkers and as tools in IVEP is particularly promising, especially considering their ability to deliver bioactive molecules for targeted cell modulation. However, challenges such as high production costs and limited scalability for large-scale use must be addressed. Continued research is crucial to overcome these limitations and to establish standardized, safe, and efficient protocols for their application in reproductive technologies.
